# Spontaneous neural activity in the developing ant olfactory system

**DOI:** 10.64898/2026.10.25.761666

**Published:** 2026-10-29

**Authors:** Dominic D Frank, Daniel J. C. Kronauer

## Abstract

Spontaneous neural activity is a pillar of mammalian neurodevelopment. In mammals, developing olfactory sensory neurons (OSNs) show spontaneous activity patterns that depend on the olfactory receptor (OR) they express. This activity is necessary for proper sorting of OSN axons into olfactory bulb glomeruli. This contrasts with the simpler olfactory system of the fruit fly Drosophila melanogaster, where antennal lobe glomeruli form without functional ORs. Compared to Drosophila, ants have a vastly expanded olfactory system and, unlike Drosophila, require functional ORs for proper development. This raises the possibility that ants, like mammals, rely on spontaneous neural activity. To test whether developing ant OSNs are spontaneously active, we performed live calcium imaging in translucent pupae of the clonal raider ant Ooceraea biroi, using a transgenic line expressing the genetically encoded calcium indicator GCaMP6s in OSNs. Developing O. biroi OSNs exhibit spontaneous calcium transients from the earliest stages of glomerulus formation, and features of this activity change over the course of metamorphosis. During early stages, activity is faster, varies widely between cells, and is uncorrelated across the OSN population. By mid-metamorphosis, activity has slowed, with longer calcium events and fewer peaks per cell. As pupae approach eclosion, a new mode appears, with rhythmic, sawtooth-shaped calcium events that synchronously engage OSNs in both antennae. This stage-dependent spontaneous activity suggests that the expanded ant olfactory system may use a developmental logic more akin to mammals than to Drosophila.

## Full Text Availability

The license terms selected by the author(s) for this preprint version do not permit archiving in PMC. The full text is available from the preprint server.

